# Association between altered metabolism and genetic mutations in human glioma

**DOI:** 10.1002/cnr2.1799

**Published:** 2023-03-14

**Authors:** Hannah Pearl, Candace C. Fleischer

**Affiliations:** ^1^ College of Arts and Sciences Tufts University Medford Massachusetts USA; ^2^ Department of Radiology and Imaging Sciences Emory University School of Medicine Atlanta Georgia USA; ^3^ Department of Biomedical Engineering Georgia Institute of Technology and Emory University Atlanta Georgia USA

**Keywords:** 1p/19q, ex vivo NMR, glioblastoma, glioma, IDH mutations, metabolic reprogramming

## Abstract

**Background:**

Molecular markers for classification of gliomas include isocitrate dehydrogenase (IDH) mutations and codeletion of chromosomal arms 1p and 19q (1p/19q). While mutations in IDH enzymes result in the well‐characterized production of oncometabolite 2‐hydroxyglutarate, dysregulation of other metabolites in IDH tumors is less characterized. Similarly, the effects of 1p/19q codeletion on cellular metabolism are also unclear.

**Aim:**

This study aimed to quantify changes in tumor metabolites in human glioma tissue as a function of both IDH mutation and 1p/19q codeletion.

**Methods and Results:**

Deidentified human glioma tissue and associated clinical data were obtained from the Emory University Winship Cancer Institute tissue biobank from 14 patients (WHO grades II, III, and IV; seven female and seven male). Proton (^1^H) high‐resolution magic angle spinning (HR‐MAS) nuclear magnetic resonance (NMR) spectroscopy data were acquired using a 600 MHz Bruker AVANCE III NMR spectrometer. Metabolite concentrations were calculated using LCModel. Differences in metabolite concentrations as a function of IDH mutation, 1p/19q codeletion, and survival status were determined using Mann–Whitney *U* tests. Concentrations of alanine, glutamine, and glutamate were significantly lower in glioma tissue with IDH mutations compared to tissue with IDH wildtype. Additionally, glutamate concentration was significantly lower in glioma tissue with 1p/19q codeletion compared to intact 1p/19q. Exploratory analysis revealed alanine concentration varied significantly as a function of survival status.

**Conclusions:**

Given the emerging landscape of glioma treatments that target metabolic dysregulation, an improved understanding of altered metabolism in molecular sub‐types of gliomas, including those with IDH mutation and 1p/19q codeletion, is an important consideration for treatment stratification and personalized medicine.

## 1 INTRODUCTION

Gliomas are a heterogeneous disease comprising 80% of malignant brain tumors and accounting for most deaths resulting from primary brain tumors.^1^ In 2016, the World Health Organization (WHO) revised the guidelines for classification of gliomas to include molecular markers, for the first time, in addition to histology^2^ as histology alone is often insufficient for the development of personalized treatment.^3^ Molecular markers include, among others, isocitrate dehydrogenase (IDH) mutations and codeletion of chromosomal arms 1p and 19q (1p/19q). IDH mutations are primarily observed in low grade gliomas and high grade secondary glioblastomas.^4^, ^5^ The 1p/19q codeletion is observed primarily in oligodendrogliomas, which comprise approximately 4%–15% of all gliomas.^6^, ^7^ The WHO 2016 guidelines for classification of gliomas include three categories: IDH‐mutant with 1p/19q codeletion, IDH‐mutant without 1p/19q codeletion, and IDH‐wildtype. Gliomas with 1p/19q codeletion and without IDH mutation are rare.^8^ Given the heterogeneity of gliomas, combined with poor outcomes particularly in higher grades, a better understanding of molecular subtypes is necessary.

One hallmark of cancer, including gliomas, is metabolic reprogramming. Metabolic alterations support aberrant tumor growth, enable circumvention of immune‐mediated cell death, and allow cancer cells to adapt to metabolic stress, including hypoxia and the acidic tumor microenvironment.^9^, ^10^ The most well‐known example of metabolic reprogramming is the Warburg effect in which cancer cells have increased preference for glycolysis over oxidative phosphorylation even in the presence of oxygen and despite having functioning mitochondria.^11^ The Warburg effect is marked by an increase in both glucose uptake and production of lactate. While there is no consensus regarding the adaptive advantage this confers on the cell, several hypotheses have been proposed. It is possible a higher rate but lower yield of ATP production is advantageous when resources are limited. Additionally, the increased production of lactate may contribute to acidosis, which benefits tumor growth.^11^ In addition to glucose and lactate, other metabolites have also been shown to be dysregulated in cancer. For example, previous work by our group demonstrated dysregulation of glutamine and its associated metabolites, including alanine and glutamate, in human glioma tissue as a function of tumor grade and inflammation.^12^ Glutamine serves as a nitrogen source for synthesis of nucleic acids and other amino acids, which are used to facilitate rapid proliferation of cancer cells and to replenish the tricarboxylic acid cycle intermediates through anaplerosis.^13^ Furthermore, total *N*‐acetylaspartate + *N*‐acetylaspartate glutamate (tNAA) and total choline (tCho)—metabolites detectable with ^1^H nuclear magnetic resonance (NMR) spectroscopy—are commonly dysregulated in cancer including gliomas.^14^, ^15^

While many metabolic alterations are heterogeneous and evolve as cancer progresses, some result from known genetic alterations such as the IDH mutation.^16^ The wild‐type function of the IDH enzyme results in the conversion of isocitrate to α‐ketoglutarate. The heterozygous IDH mutation, most commonly at an arginine (R) residue (i.e., *IDH1*(R132) or *IHD2*(R172)), results in the conversion of α‐ketoglutarate to 2‐hydroxyglutarate (2‐HG), a biomarker for IDH‐mutant gliomas. The IDH1 mutation occurs in the cytoplasm and IDH2, its homolog, in the mitochondria.^5^, ^17^ As a result, multiple pathways are dysregulated, including those related to hypoxia and glutamine metabolism, to compensate for liabilities of the IDH mutation.^17^ The IDH mutation is related to oncogenesis through the production of 2‐HG which can result in DNA and histone hypermethylation; however, a precise mechanism for the role of IDH mutations in tumor progression or inhibition has yet to be elucidated.^18^ While clinical trials of inhibitors of mutant IDH enzymes in acute myeloid leukemia are being conducted,^16^, ^19^ there are currently no therapeutic targets specific to IDH‐mutant gliomas. Similarly, the 1p/19q codeletion was first reported in 1994, but its biological effects remain unclear, and the associated metabolic dysregulation is understudied.^3^ We hypothesize molecular subtypes of gliomas are associated with distinct metabolic changes beyond the well‐characterized presence of 2‐HG. The goal of this study was to characterize metabolite levels in human glioma tissue, as a function of both IDH status and 1p/19q codeletion, using ex vivo ^1^H‐NMR spectroscopy,^12^, ^14^

## 2 METHODS

### 2.1 Human glioma tissue

This study was not classified as human subjects research or clinical investigation, and was determined to be exempt by the Emory University Institutional Review Board. All samples were existing tissue specimens and no identifying information regarding the samples was provided to researchers. Deidentified human glioma tissue and associated clinical data were obtained from the Emory University Winship Cancer Institute tissue biobank from 14 patients (WHO grades II, III, and IV; seven female and seven male). Inclusion criteria were patients ≥18 years old, with a diagnosis of diffuse astrocytoma, infiltrating astrocytoma, oligodendroglioma, anaplastic oligodendroglioma, anaplastic astrocytoma, glioblastoma, high grade astrocytoma, or high grade glioma. There was no attrition; NMR spectra were acquired from all 14 samples that met the inclusion criteria.

### 2.2 ^1^H high resolution magic angle spinning nuclear magnetic resonance spectroscopy

^1^H high resolution magic angle spinning (HR‐MAS) NMR spectra were acquired as previously described.^12^ Briefly, glioma tissue (~10–15 mg) was placed into an 80 μL HR‐MAS disposable insert (Bruker, B4493) inside a 4 mm zirconium oxide HR‐MAS rotor (Bruker, H14355). NMR experiments were performed at 4°C using a 600 MHz Bruker AVANCE III NMR spectrometer with the Carr‐Purcell‐Meiboom‐Gill (cpmgr1d) pulse sequence and a pre‐saturation water suppression pulse. Parameters were as follows: MAS speed = 4000 Hz; complex data points = 16384; spectral bandwidth = 8013 Hz; N = 512; and flip angle = 90°. Brain metabolite concentration ratios were calculated using LCModel, a user‐independent, frequency‐domain method for spectral quantification.^20^ Spectra were analyzed between 3.85 and 0.2 ppm using a 26‐metabolite set with MR‐detectable metabolites present in gliomas. Spectral analysis was performed in random order by a researcher blinded to the subject groups to mitigate bias, and LCModel analysis was automated. Metabolite concentrations (alanine, glutamine, glutamate, tNAA, and tCho) were normalized to total creatine (tCr).

### 2.3 Statistical analysis

Statistical analysis was performed in R 4.0.1. Differences in metabolite concentrations between groups as a function of IDH mutation, 1p/19q codeletion, and survival status were evaluated using Mann–Whitney *U* tests. Given the small sample size, sex as a biological variable was not evaluated; however, sex, race, and ethnicity for all samples is included in Table S1. *P*‐values for all metabolites within groups (IDH mutation or 1p/19q codeletion) were corrected for the false discovery rate (FDR), and both uncorrected and FDR‐corrected *p*‐values are reported. Significance was determined by *p* ≤ .05. Values are reported as the median (interquartile range) unless otherwise noted.

## 3 RESULTS

**FIGURE 1:**
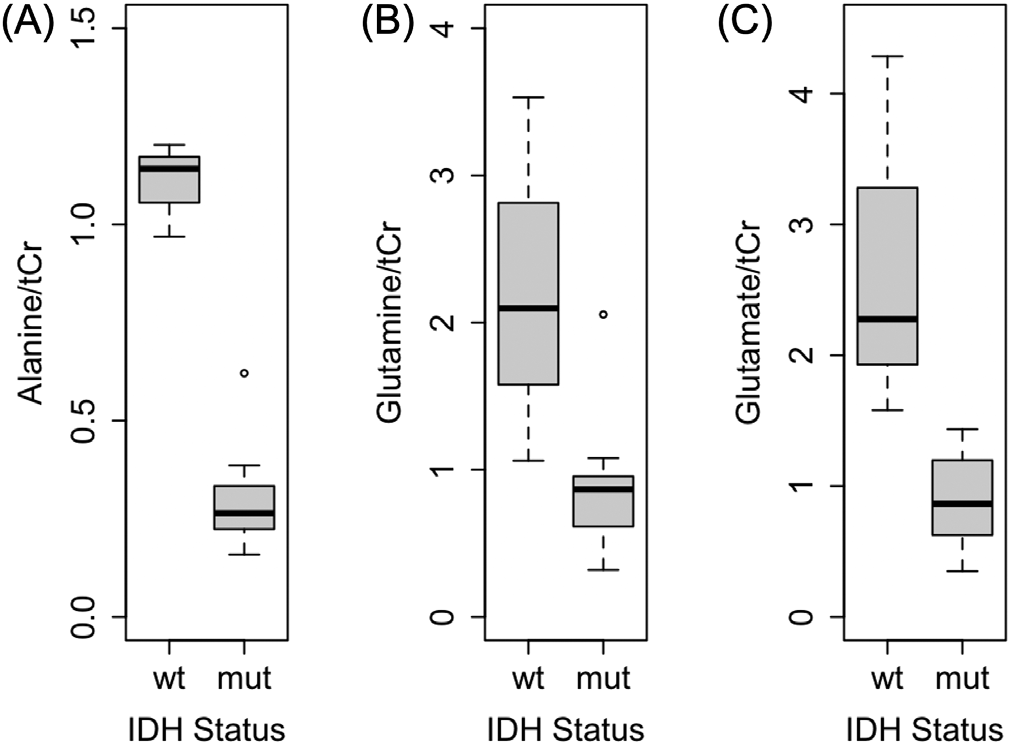
Tumor metabolites were significantly lower in glioma tissue with isocitrate dehydrogenase (IDH) mutation compared to tissue with IDH wildtype, including (A) alanine, (B) glutamine, and (C) glutamate. tCr, total creatine; mut, IDH‐mutant; wt, IDH‐wildtype

**FIGURE 2:**
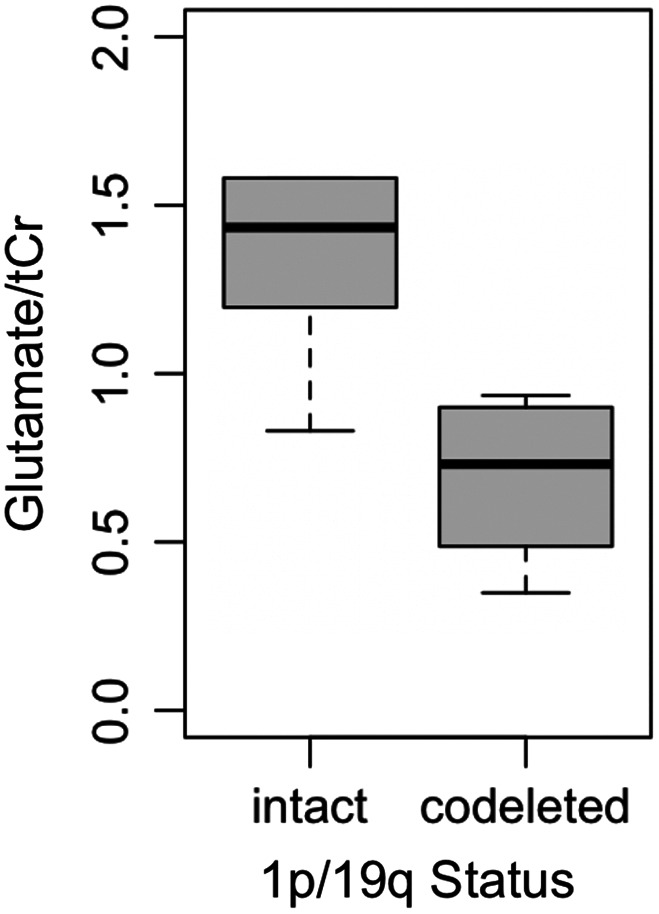
Glutamate concentration was significantly lower in glioma tissue with 1p/19q codeletion compared to intact 1p/19q. tCr, total creatine

**FIGURE 3:**
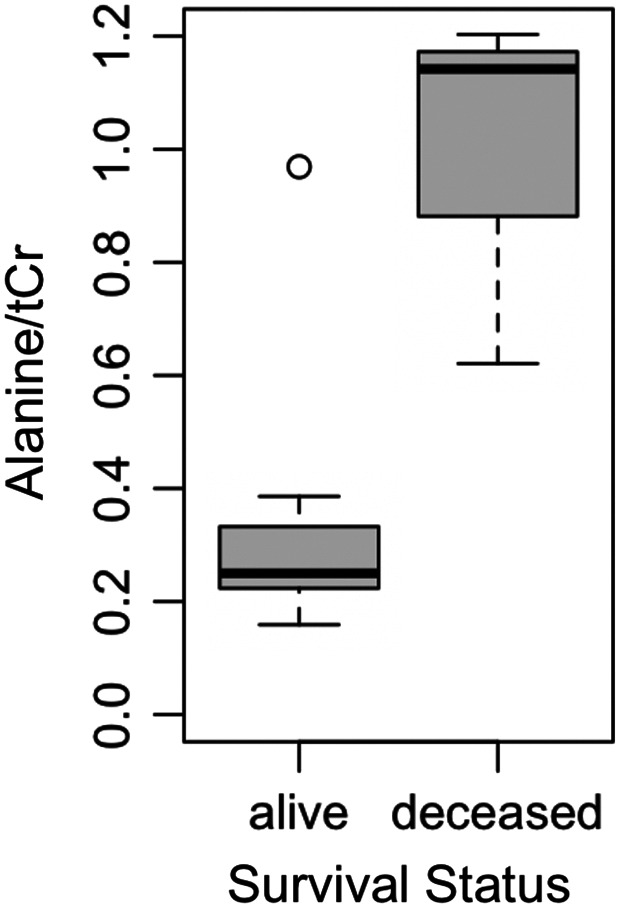
Alanine is significantly higher in samples from deceased patients compared to those who are still alive. tCr, total creatine

Patient characteristics for all glioma samples are shown in Table 1. Alanine and glutamate concentrations were both significantly lower in IDH‐mutated glioma tissue compared to IDH‐wildtype (Table 2, Figure 1). Glutamine levels were significantly lower in IDH‐mutant glioma tissue (*p* = .036), but after FDR correction, the difference was not significant (*p*_FDR_ = .061). The remaining metabolites did not differ significantly according to IDH status. Glutamate concentrations were significantly lower in glioma tissue with 1p/19q codeletion compared to intact samples (*p* = .032) but did not survive FDR correction (*p*_FDR_ = 0.159; Table 3, Figure 2). The remaining metabolites did not differ significantly between 1p/19q codeleted and intact samples (*p* > .05). Exploratory analysis revealed alanine concentration is significantly lower in patients who were alive at date of last contact (minimum time was 6.6 months) compared to deceased individuals (*p* = .033; Figure 3).

**TABLE 1:** Characteristics of glioma samples

Sample	WHO Grade	IDH Status	1p/19q	Survival status
1	III	Mutant (*IDH1*)	Codeleted	Alive
2	III	Mutant (*IDH2*)	Codeleted	Alive
3	IV	Mutant (*IDH1*)	Intact	Deceased
4	II	Mutant (*IDH1*)	Unknown	Alive
5	IV	WT	Intact	Deceased
6	II	Mutant (*IDH1*)	Codeleted	Alive
7	III	Mutant (*IDH1*)	Unknown	Alive
8	IV	WT	Unknown	Deceased
9	IV	WT	Intact	Alive
10	III	Mutant (*IDH1*)	Intact	Alive
11	II	Mutant (*IDH1*)	Intact	Unknown
12	II	Mutant (*IDH1*)	Intact	Unknown
13	III	Mutant (*IDH1*)	Codeleted	Alive
14	II	Mutant (*IDH1*)	Codeleted	Alive

Abbreviations: IDH, isocitrate dehydrogenase; WT, wildtype; WHO, World Health Organization.

**TABLE 2:** Differences in tumor metabolite concentrations as a function of IDH status

Metabolite^a^	IDH WT	IDH mut	*U* ^b^	*p*	*p*_FDR_ ^c^
Alanine	1.14 (.12)	0.26 (0.08)	0	**0.012**	**0.030**
Glutamine	2.10 (1.23)	0.87 (0.34)	0	**0.036**	0.061
Glutamate	2.28 (1.35)	0.87 (0.48)	0	**0.007**	**0.030**
tNAA	0.596^d^	0.71 (0.60)	3	0.889	>0.99
tCho	0.90 (0.38)	0.63 (0.30)	8	0.287	>0.99

*Note*: Bolded values indicate significant *p*‐values (*p* ≤ .05).

Abbreviations: IDH, isocitrate dehydrogenase; mut, mutant; tNAA, total *N*‐acetylaspartate + *N*‐acetylaspartate glutamate; tCho, total choline; WT, wildtype.

^a^ Metabolite concentrations were normalized to total creatine (tCr).

^b^ Group‐wise comparisons were performed using Mann–Whitney *U* tests.

^c^ *p*‐values were corrected for the false discovery rate (FDR).

^d^ Only one sample had detectable tNAA concentration.

**TABLE 3:** Differences in tumor metabolite concentrations as a function of 1p/19q codeletion

Metabolite^a^	Intact	Codeleted	*U* ^b^	*p*	*p*_FDR_ ^c^
Alanine	0.62 (0.69)	0.25 (0.11)	3	0.250	>0.99
Glutamine	1.06 (1.19)	0.73 (0.42)	5	0.286	>0.99
Glutamate	1.43 (0.38)	0.73 (0.41)	2	**0.032**	0.159
tNAA	0.76 (0.51)	0.81 (0.38)	6	>0.99	>0.99
tCho	0.83 (0.29)	0.70 (0.16)	8	0.476	>0.99

*Note*: Bolded values indicate significant *p*‐values (*p* ≤ .05).

Abbreviations: tNAA, total *N*‐acetylaspartate + *N*‐acetylaspartate glutamate; tCho, total choline.

^a^ Metabolite concentrations were normalized to total creatine (tCr).

^b^ Group‐wise comparisons were performed using Mann–Whitney *U* tests.

^c^ *p*‐values were corrected for the false discovery rate (FDR).

Among IDH‐mutant glioma tissue, one outlier (sample 3) in alanine concentration was observed (Figure 1A), representing the only patient known to be deceased among those with IDH mutation (Table 1). Despite containing higher alanine levels compared to other IDH‐mutant samples,^1^, ^2^, ^4^, ^6^, ^7^, ^10^, ^11^, ^12^, ^13^, ^14^ alanine concentration was still lower than all IDH‐wildtype samples.^5^, ^8^, ^9^ This patient also had a longer survival time (75.2 months) and lower alanine levels compared to the two other deceased patients for whom alanine was quantified (12.6 (sample 5) and 0.1 (sample 8) months).

## 4 DISCUSSION

Metabolic dysregulation is well‐documented in gliomas; however, the association between genetic mutations and tumor metabolism is less characterized. We observed lower alanine concentrations in glioma tissue with IDH mutation compared to IDH‐wildtype. While a mechanistic link between altered alanine metabolism and IDH status is unknown, Firdous et al determined alanine is a key metabolite for discriminating between glioma and non‐glioma tissue using untargeted NMR‐based metabolomics and machine learning.^21^ Alanine metabolism has been investigated as a potential target for therapeutic treatment of gliomas. Brennan et al investigated the effects of gliotoxins on alanine metabolism and observed reduced flux from [3‐^13^C] alanine to glutamate,^22^ as well as significantly less [3‐^13^C] alanine produced from [1‐^13^C] glucose.^23^ While the mechanism by which the IDH mutation might affect alanine metabolism is not entirely clear, alanine is also involved in ammonia transfer which is necessary for glutamate/glutamine cycling in the brain.^24^ Specifically, alanine aminotransferase transfers an amino group from alanine to α‐ketoglutarate, resulting in the production of glutamate and pyruvate.^25^ It is reasonable that dysregulation in glutamine and glutamate pathways may also affect alanine levels, as lower levels of glutamine and glutamate may result in a decreased need for alanine and subsequent decrease in alanine levels. This may account for our finding of decreased alanine levels in tissue with IDH mutation. Jalbert et al also observed different metabolic profiles in IDH‐mutant gliomas compared to IDH‐wildtype gliomas using ex vivo NMR. While Jalbert et al did not observe significant differences in alanine concentration in IDH‐mutant compared to IDH‐wildtype gliomas, IDH mutation is primarily observed in lower grade gliomas and they did report lower alanine levels in tissue with lower WHO grade.^14^ This is consistent with our previous work, which demonstrated significantly lower tumor alanine concentration with lower WHO grade.^12^ While WHO grade may partially account for our findings, IDH status likely also plays an independent role. Among all deceased patients with grade IV glioblastoma, the single patient with IDH mutation had markedly lower alanine levels compared to individuals with IDH‐wildtype. Increased alanine may also be an indicator of later disease stage, as this patient also had a longer survival time and lower alanine levels compared to other patients who were deceased.

Changes in glutamine and glutamate complement our previous findings of dysregulated glutamine‐associated metabolites as a function of tumor grade and inflammation.^26^ Our current results are consistent with previous studies showing decreased glutamate levels in IDH‐mutant compared to IDH‐wildtype cells.^27^ McBrayer et al showed the oncometabolite 2‐HG inhibits branched‐chain amino acid transaminase, the enzyme responsible for conversion of branched‐chain amino acids to glutamate, leading to decreased levels of glutamate.^28^ Additionally, glutamate dehydrogenase (GDH), which converts glutamate to α‐ketoglutarate, compensates for deficiencies of IDH‐mutant cells.^29^ The need for increased levels of glutamate as a substrate for GDH is met, in part, by glutaminase, the enzyme that converts glutamine to glutamate during glutaminolysis.^17^, ^30^, ^31^ Ohka et al observed decreased glutamine and glutamate levels resulting from upregulation of glutaminolysis in cells expressing IDH1 mutation.^32^ Further investigation into the mechanistic relationship between glutamine, glutamate, and IDH mutation may be warranted.

Little is known about the biological effects of 1p/19q codeletion. Metabolomic studies have yielded conflicting results.^3^ Notably, one *in vivo* study by Branzoli et al observed elevated levels of cystathionine in gliomas with 1p/19q codeletion. The authors suggest a possible mechanism related to the loss of two enzymes located on chromosomal arm 1p, phosphoglycerate dehydrogenase and cystathionine gamma‐lyase.^3^ To our knowledge, no study has shown dysregulated glutamate metabolism as a result of 1p/19q codeletion. Interestingly, there was one IDH‐mutant tumor sample containing high levels of alanine that did not possess 1p/19q codeletion, while the majority of IDH‐mutant samples were also 1p/19q codeleted. The three molecular subtypes of gliomas are IDH‐mutant with 1p/19q codeletion, IDH‐mutant without 1p/19q codeletion, and IDH‐wildtype. Among these, IDH‐mutant with 1p/19q codeletion has the most favorable prognosis, followed by IDH‐mutant without 1p/19q codeletion. IDH‐wildtype gliomas have the worst prognosis among these molecular phenotypes.^4^ Examining metabolic phenotypes in light of emerging molecular phenotypes is an important area for future investigation.

Current clinical methods for diagnosis of brain tumors and treatment planning require tissue biopsy, which is invasive and can cause postoperative complications, such as hemorrhage, in a small percentage of patients.^33^ Previous studies have shown the clinical utility of non‐invasive ^1^H magnetic resonance spectroscopy (MRS) in characterizing tumor grade and type^34^; however, aside from 2‐HG, there is a lack of noninvasive imaging biomarkers that can be used to distinguish between tumor type, aid in treatment planning, and monitor response to treatment.^35^ The advantage of ex vivo NMR studies, including the present pilot study, is the natural translation to MRS, as in vivo MR scanners used to acquire MRS data rely on the same physical principles and similar hardware as analytical NMR instrumentation. We acknowledge a limitation of the study is the sample size, and replication in a larger cohort is necessary. While the *in situe* design of this study allowed for metabolite quantification in the original tumor microenvironment, gliomas exhibit a high level of metabolic heterogeneity across different regions of the same tumor.^16^ Future studies using whole‐brain MRS will facilitate evaluation of heterogeneous metabolic patterns toward targeted treatment.^36^ Additionally, NMR and MRS both facilitate simultaneous detection of 10–20 brain metabolites; however, a subset of metabolites, based on prior work, was assessed in this pilot study due to limited statistical power. Future studies will benefit from a complete metabolic or metabolomic analysis in a larger cohort. While the observed *IDH1* mutation was typically arginine replaced with histidine at codon 132 (encoding *IDH1*(R132H)) and the *IDH2* mutation was arginine replaced with methionine at codon 172 (encoding *IDH2*(R172M)), the type of IDH mutation was not evaluated explicitly as this information was not available for all samples. Determining genetic profiles in both histological tissue and peripheral blood are important goals for future work. Finally, metabolite levels were assessed in IDH‐mutant and IDH‐wildtype glioma tissue, but control tissue was absent given limitations in obtaining healthy brain tissue.

## 5 CONCLUSION

Advances in recent years have revealed gliomas are highly heterogeneous, and subtypes exhibit distinct metabolic landscapes and different responses to treatment.^17^ The results of the current study represent an important first step in identifying associations and interactions between metabolic phenotypes and molecular (genetic) subtypes in gliomas, which are commonly treated separately but likely inextricably linked in the tumor microenvironment. A better understanding of molecular markers, including IDH mutations and 1p/19q codeletion, and the affected pathways is necessary for improved disease stratification, prognosis, and development of therapeutic targets.

## AUTHOR CONTRIBUTIONS

**Hannah Pearl:** Data curation (supporting); formal analysis (lead); investigation (equal); visualization (lead); writing – original draft (lead). **Candace C. Fleischer:** Conceptualization (lead); data curation (lead); investigation (equal); supervision (lead); writing – review and editing (lead).

## CONFLICT OF INTEREST STATEMENT

The authors declare no conflict of interest.

## ETHICS STATEMENT

This study was not classified as human subjects research or clinical investigation, and was determined to be exempt by the Emory University Institutional Review Board.

## Supporting information


**Table S1:** Compiled subject‐level raw data used for analysis

## Data Availability

All data is available in Table S1. Raw data is available upon request from the corresponding author upon execution of a data sharing agreement.
